# Towards higher sensitivity and stability of axon diameter estimation with diffusion‐weighted MRI

**DOI:** 10.1002/nbm.3462

**Published:** 2016-01-08

**Authors:** Farshid Sepehrband, Daniel C. Alexander, Nyoman D. Kurniawan, David C. Reutens, Zhengyi Yang

**Affiliations:** ^1^ Centre for Advanced ImagingThe University of QueenslandBrisbaneAustralia; ^2^ Queensland Brain InstituteThe University of QueenslandBrisbaneAustralia; ^3^ Department of Computer Science & Centre for Medical Image ComputingUniversity College LondonLondonUK; ^4^ School of Information Technology and Electrical EngineeringThe University of QueenslandBrisbaneAustralia; ^5^ Brainnetome Center, Institute of AutomationChinese Academy of SciencesBeijingChina; ^6^ Faculty of Information EngineeringSouthwest University of Science and TechnologyMianyangChina

**Keywords:** diffusion‐weighted MRI, ActiveAx, ultra‐high gradient strength, axon diameter index, histological validation, electron microscopy, mouse corpus callosum

## Abstract

Diffusion‐weighted MRI is an important tool for *in vivo* and non‐invasive axon morphometry. The ActiveAx technique utilises an optimised acquisition protocol to infer orientationally invariant indices of axon diameter and density by fitting a model of white matter to the acquired data. In this study, we investigated the factors that influence the sensitivity to small‐diameter axons, namely the gradient strength of the acquisition protocol and the model fitting routine. Diffusion‐weighted *ex. vivo* images of the mouse brain were acquired using 16.4‐T MRI with high (*G*_max_ of 300 mT/m) and ultra‐high (*G*_max_ of 1350 mT/m) gradient strength acquisitions. The estimated axon diameter indices of the mid‐sagittal corpus callosum were validated using electron microscopy. In addition, a dictionary‐based fitting routine was employed and evaluated. Axon diameter indices were closer to electron microscopy measures when higher gradient strengths were employed. Despite the improvement, estimated axon diameter indices (a lower bound of ~ 1.8 μm) remained higher than the measurements obtained using electron microscopy (~1.2 μm). We further observed that limitations of pulsed gradient spin echo (PGSE) acquisition sequences and axonal dispersion could also influence the sensitivity with which axon diameter indices could be estimated. Our results highlight the influence of acquisition protocol, tissue model and model fitting, in addition to gradient strength, on advanced microstructural diffusion‐weighted imaging techniques. © 2016 The Authors. NMR in Biomedicine published by John Wiley & Sons Ltd.

**Abbreviations used**
*ActiveAx‐D*: *ActiveAx using dictionary‐based parameter extraction*
*AMICO*: *accelerated microstructure imaging via convex optimisation*
*ANOVA*: *analysis of variance; DTI, diffusion tensor imaging*
*EM*: *electron microscopy*
*MCMC*: *Markov chain Monte Carlo*
*MMWMD*: *minimal model of white matter diffusion*
*NODDI*: *neurite orientation distribution and density imaging*
*OGSE*: *oscillating gradient spin echo*
*PFA*: *paraformaldehyde*
*PBS*: *phosphate‐buffered saline*
*PGSE*: *pulsed gradient spin echo*
*SNR*: *signal‐to‐noise ratio.*

## Introduction

Advances in diffusion‐weighted MRI over the past 19 years have provided *in vivo* non‐invasive estimates of axon morphometry 1, 2, 3, 4, 5, 6, 7, 8. Information from axon morphometry may contribute to a better understanding of neural conduction, which underpins connectivity within the nervous system, its development 9, 10 and its contribution to the pathophysiology of disorders, such as multiple sclerosis and autism 11, 12, 13.

ActiveAx is a diffusion‐weighted MRI model‐based technique that provides an orientationally invariant ‘axon diameter index’, a summary statistic of the axon diameter distribution, using acquisition protocols that are feasible for *in vivo* human imaging 3. This technique models the geometry of tissue microstructure and fits the model to diffusion‐weighted measurements of different encoding properties (e.g. duration, directions and strengths). The model assumes that the signal attenuation during the diffusion‐encoding gradient originates from the sum of water displacements in different tissue media, such as intra‐ and extra‐axonal spaces. The patterns of water displacement differ across media as a result of the morphological characteristics of the tissue microstructure. ActiveAx fits a minimal model of white matter diffusion (MMWMD) to the diffusion‐weighted data, in which the intra‐axonal space is defined by a model of restricted diffusion of water trapped in a bundle of cylinders with equal radii, and the extra‐axonal space is defined by a model of hindered water displacement, with a tortuosity hindrance in the direction perpendicular to the axons. Additional compartments, such as cerebrospinal fluid and stationary water, can also be added to optimise the method for *in vivo* or *ex. vivo* imaging 3, 14.

It is a challenging task to obtain high sensitivity and stability in axon diameter index estimates. Sensitivity is mainly limited by the scanner's gradient strength and by the type of pulse sequence 7. For example, it has been shown that the sensitivity with which the small axon diameter can be measured improves significantly by moving from the gradient strength of current clinical scanners (~60 mT/m) to the gradient strength used in the human connectome project (300 mT/m) 6, 7. The stability of parameter estimates with ActiveAx is affected by the non‐linear parameter fitting procedure employed. The objective functions for these fitting procedures often have many local minima, rendering the determination of the global minimum challenging and time consuming. Recently, Daducci *et al*. 15 have proposed a framework for accelerated microstructure imaging via convex optimisation (AMICO), which linearises the fitting procedure, dramatically reducing the computation time. They also observed that AMICO yields a higher stability of axon diameter indices by avoiding local minima.

In this work, we aimed to tackle the challenge of increasing the sensitivity and stability of axon morphometry, specifically estimates of axon diameter indices, by optimising the acquisition and methods of parameter estimation. In brief:

- In the acquisition stage, a pulse sequence with an ultra‐high gradient strength was designed, in which a high‐field, small‐bore scanner was pushed to its limits in terms of the gradient strength. A pulsed gradient spin echo (PGSE) protocol with *G*_max_ of 1350 mT/m (maximum *b* value of 105 000 s/mm^2^) was tested in our study. An additional PGSE protocol with *G*_max_ of 300 mT/m (maximum *b* value of 9500 s/mm^2^), previously described in ref. 7, was used to assist the comparison and evaluation of the first protocol. Both protocols were optimised for high sensitivity to mouse callosal axons using the framework explained in ref. 16.
- In the parameter extraction stage, a dictionary‐based routine was employed, similar to AMICO, with a few modifications. We generated a dictionary for each voxel, informed by the data, in which some characteristics of the tissue and signal, such as fibre orientation and signal‐to‐noise ratio (SNR), were pre‐specified, allowing the dictionary to reflect only the main parameters of interest. The key differences of our parameter extraction technique from AMICO are in model assumptions. AMICO uses a tensor model for prior estimation of fibre orientation and a Gaussian distribution to model the noise distribution. We used MMWMD for the prior estimation of fibre orientation and the Rician model as the noise distribution model, sacrificing computational speed for improved sensitivity and stability in the estimation of the axon diameter index.

We scanned the brain of a sacrificed mouse with a 16.4‐T scanner, and validated the results with post‐scan electron microscopy (EM) in subregions of the mouse corpus callosum. Axon diameter indices obtained from the acquisition protocol with *G*_max_ = 1350 mT/m were significantly lower than those obtained from the acquisition protocol with *G*_max_ = 300 mT/m (~2 μm for the former and ~3 μm for the latter). In addition, the parameter extraction routine employed resulted in increased sensitivity and stability of axon diameter index estimates. However, despite the improvement, the estimated axon diameter indices remained higher than the EM results (~1.2 μm), as in previous studies 3, 7. To understand the reason for this overestimation, we investigated three possible causes.

1. Time‐dependent diffusion in extra‐axonal water 17. It has been suggested recently that, for short diffusion times, the resulting values are dominated by non‐Gaussian diffusion in extra‐axonal water, biasing estimates of the axon diameter 18. To minimise this effect, measurements with low *b* values were removed prior to axon diameter index estimation.
2. PGSE pulse sequence limitation. The same acquisition protocol as used in our *ex. vivo* scan was employed in simulations of the signal change expected for a wide range of axon diameter indices. The axonal compartment of MMWMD was used to model intra‐axonal diffusion perpendicular to the axon axis 19.
3. Presence of axonal dispersion. ActiveAx assumes a bundle of aligned axons, which, in the presence of axonal dispersion, could bias estimates of the axon diameter index. The presence of axonal dispersion was investigated by measuring the fractional anisotropy from diffusion tensor imaging (DTI) 20, 21 and the orientation dispersion index from neurite orientation distribution and density imaging (NODDI) 20, 21. In addition, axonal dispersion in histological images was assessed qualitatively.

We observed that, by removing measurements with low *b* values, the axon diameter indices were slightly closer to EM estimates, but this change was not statistically significant. The *b* value sampling rate used here was not sufficiently dense to allow rigorous assessment of the effect of time‐dependent diffusion in extra‐axonal water on axon diameter indices. This effect requires further investigation, as the current models do not reveal the complexity of white matter microstructure, for example by neglecting restrictions arising from unmyelinated axons and cellular processes. We also observed that limitations of the PGSE pulse sequence and axonal dispersion could contribute to the overestimation of axon diameter, highlighting the need for more sensitive pulse sequences and more comprehensive tissue models as the focus of future directions.

## Method

We used EM images as the ground truth to validate our estimates of the axon diameter index from diffusion‐weighted imaging. The high magnification of EM images allows an accurate estimation of the diameter of callosal axons in the transverse plane (mid‐sagittal section). We used a fixation technique that has been demonstrated to produce almost no tissue shrinkage; techniques used to generate previously published estimates of axon diameter have been associated with 40–70% shrinkage 22. The inner diameters of axons were measured from EM images to obtain the axon diameter distribution in each region. Axon diameter indices were then obtained by calculating the mean axon diameter weighted by volume, as explained in Equations [2] and [3].

The sensitivity and stability of the estimated axon diameter indices from different datasets and techniques were investigated. Sensitivity was evaluated by comparing the mean axon diameter indices within specific regions of the callosum against ground truth axon diameter indices from EM (see ‘Axon diameter index’ section below). Stability was evaluated by the variance in the estimated axon diameter indices in each callosal region. Specifically, we define ‘sensitivity’ as |*α*_EM_ – *μ*| and ‘specificity’ as $\sqrt{\frac{1}{N-1}\sum_{i=1}^{N}{\left|\alpha_{i}-\mu \right|}^{2}}$, where *α* and *α*_EM_ are the axon diameter indices from diffusion MRI and EM, respectively, and *μ* is the mean of the axon diameter indices from diffusion MRI in a given region consisting of *N* voxels.

### Animal housing and preparation

All procedures were performed with the approval of The University of Queensland Animal Ethics Committee under the guidelines of the National Health and Medical Research Council of Australia. One 8‐week‐old male C57Bl/6 J mouse was reared and housed at the Queensland Brain Institute Animal Facility, The University of Queensland. The animal was housed in a 12‐h light/dark cycle with free access to food and water.

We scanned a mouse brain, as mouse models of neurological diseases are commonly used because of the availability of relevant mouse mutants and of gene targeting technology 23. The adult mouse was anaesthetised with an intraperitoneal injection of approximately 8–9 mg/mL sodium pentobarbitone (Lethabarb™; Virbac, Milperra, NSW, Australia) and then transcardially perfused with 0.9% saline solution [0.9% *w*/*v* NaCl in MilliQ™ (Millipore, Bayswater, VIC, Australia) water] for 5 min, followed by 4% paraformaldehyde (PFA) (4% PFA *w*/*v*; ProSciTech, Townsville, Qld, AU) with 0.2% Magnevist® (1 mm gadopentetate dimeglumine, Bayer, Pymble, NSW, Australia) in phosphate‐buffered saline (PBS: 137 mm NaCl, 10 mm Na_2_HPO_4_, 1.8 mm KH_2_PO_4_, 2.7 mm KCl, pH 7.4) for 10 min. The steps for fixation and storage are similar to those described in ref. 24, previously optimised for diffusion‐weighted MRI studies. The brain was post‐fixed in 4% PFA with 0.2% Magnevist in PBS and stored at 4 °C. The brain was then incubated in PBS and 0.2% Magnevist for 4 days to remove the PFA prior to MRI. A Magnevist concentration of 0.2% and incubation for 4 days were found to be optimal for obtaining good SNR and contrast for *ex. vivo* brain imaging 25. A high concentration of Magnevist can influence the estimation of the extra‐axonal water diffusivity 26 and fraction, but, at the concentration used in this study, Magnevist does not affect the diffusion properties, such as the apparent diffusion coefficient and fractional anisotropy 26. The calculated water: Magnevist ratio in white matter is around 1: 5500 with a very short interaction of 1 ns between the two molecules and a radius of separation between the two molecules of 0.1–1 nm 27, 28.

### Diffusion‐weighted MRI

The brain was placed in Fomblin (Solvay Solexis, Milan, Italy) and imaged on a 16.4‐T (89‐mm) Bruker micro‐imaging system (Bruker Biospin, Karlsruhe, Germany) using a 15‐mm SAW coil with Micro2.5 gradient (M2M Imaging, Cleveland, OH, USA). Three mid‐sagittal slices were scanned with an in‐plane resolution of 100 × 100 μm^2^ and slice thickness of 300 μm (only the most central slice was used). Two PGSE acquisition protocols were used: (1) with a maximum gradient strength of 300 mT/m, as suggested in ref. 7; and (2) with a maximum gradient strength of 1350 mT/m, the highest feasible for our scanner based on the protocol optimisation criterion. For each protocol, multi‐shell data were acquired using a two‐dimensional diffusion‐weighted spin‐echo sequence, which were optimised as explained in refs. 3, 16. The protocols are shown in Table 1. When optimising the PGSE acquisition sequence with the ultra‐high gradients, the maximum gradient strength was constrained based on the scanner's maximum capability, i.e. 95% of the scanner's power. Optimally ordered gradient directions with electrostatic energy minimisation were obtained using the Camino software package 29, 30. Data are made available here (https://github.com/sepehrband/AxonDiameter).

**Table 1:** Optimised *ex. vivo* ActiveAx protocols using the pulsed gradient spin echo (PGSE) sequence of the studied protocols

*n*	|*G*| (mT/m)	*δ* (ms)	*Δ* (ms)	*b* (s/mm^2^)	*t*_d_ (ms)	1/*q* (μm)	TE (ms)
3‐shell							
120	300	5.6	12.1	2081	10.2	13.9	35
120	220	7.0	20.4	3080	18.1	15.2	35
120	300	10.5	16.9	9542	13.4	7.4	35
5‐shell							
60	1107	1.1	28.4	2974	28.0	19.3	35
60	1227	2.3	7.0	3597	6.3	8.3	35
60	464	6.3	23.0	12693	20.9	8.1	35
60	509	5.6	23.7	12706	21.8	8.2	35
120	1350	8.6	13.6	104870	10.8	2.0	35

*t*_d_ = *Δ* – *δ*/3 (s) and *b* = (2π*q*)^2^ *t*_d_ (s/m^2^), where *q* = (2π)^−1^ *γδG* (m^−1^) and *γ* = 2π × 42.57 × 10^6^ (rad/s/T).

Other information: Number of excitations  = 1; TR = 750 ms; total scan time of each dataset, 7 h, 37 min and 30 s.

We generated a third dataset from data acquired with the second PGSE acquisition protocol described above by removing the last shell (with *b* = 105 000 s/mm^2^), keeping the first four shells. This was performed to mitigate the effects of the low SNR of the last shell and the possible influence of regions of measurement space for which a simple model of white matter breaks down (e.g. the presence of fibre undulation or water exchange between compartments, which are not included in MMWMD). For simplicity, these datasets are referred to as three‐shell, four‐shell and five‐shell datasets (Table 1). It should be noted that the four‐shell data, being a subset of an optimised protocol, should not be considered as an optimised protocol *per se*.

**Axon diameter index**

The model for intra‐axonal space is the Gaussian phase distribution approximation 31 of the signal from particles trapped inside a cylinder 19, 32. In the wide‐pulse limit, the signal of water trapped between cylindrical walls can be written as follows 33:

(1) $$\ln S=-\frac{R^{4}\gamma^{2}G^{2}}{D}\frac{7}{96}\left(2\delta -\frac{99}{112}\frac{R^{2}}{D}\right)$$

where *R* is the axon radius. Therefore, the signal attenuation is proportional to the diameter to the power of 4. In the wide‐pulse limit, voxel‐averaging measurements weight the axon diameter, and the axon diameter index, a summary statistic of the axon diameter distribution, can be defined as 18:

(2) Axondiameterindexwide‐pulseα=⟨a6⟩⟨a2⟩14

where *a* is the inner diameter of individual axons. For the short‐pulse limit, the axon diameter index is defined as 18:

(3) Axondiameterindexshort‐pulseα=⟨a4⟩⟨a2⟩12

### Histological imaging

The brain was sectioned sagittally at a thickness of 50 μm using a vibratome after imaging. The sections were placed in 12‐well plates containing PBS with sodium azide and stored at 4 °C. Two mid‐sagittal sections were selected. Myelin staining and light microscopy were performed on Section 1. EM imaging was performed on Section 2 on subsections of the corpus callosum.

Section 1 was mounted on Superfrost® slides and allowed to dry overnight. Myelin staining was performed according to the manufacturer's protocol (Black‐Gold II myelin staining kit, AG105, Merck Millipore, Darmstadt, Germany). Bright‐field images of myelin‐stained sections were obtained using a (Zeiss, North Ryde, NSW, Australia) upright bright‐field slide scanner at a magnification of × 40. An AxioCam HRC digital camera (Zeiss, North Ryde, NSW, Australia) and AxioVision software (Zeiss, North Ryde, NSW, Australia) were used to capture and store the image.

On Section 2, the corpus callosum was isolated and samples of the genu, body and splenium were separated. Sample preparation was carried out according to the methods described in ref. 34. After polymerisation of the resin blocks, sections were cut on a UC6 ultra‐microtome (ultracut S, Reichert, Leica, kista, Sweden) at 60 nm, and imaged at ×5000 in a transmission electron microscope at 80 kV (JEM 1011, Jeol, Tokyo, Japan) at the Centre for Microscopy and Microanalysis of The University of Queensland. Images were captured with an Olympus Morada (Eagle Farm, QLD, Australia) digital camera.

The estimation of the axon diameters from EM was performed in MATLAB® software, version R2013a (Mathworks, Natick, MA, USA). Each axon was manually selected by drawing a circle on its transverse section (20 128 axons were segmented). The basic statistics of the axon diameters were then calculated. One‐way analysis of variance (ANOVA) with Tukey–Kramer *post hoc* correction was performed to evaluate the mean differences across subregions of the corpus callosum. *P* < 0.01 was taken to be statistically significant. The axon diameter indices were then calculated from the axon diameter distribution using Equations [2] and [3].

### Axon diameter index estimation with ActiveAx

**Figure 1:**
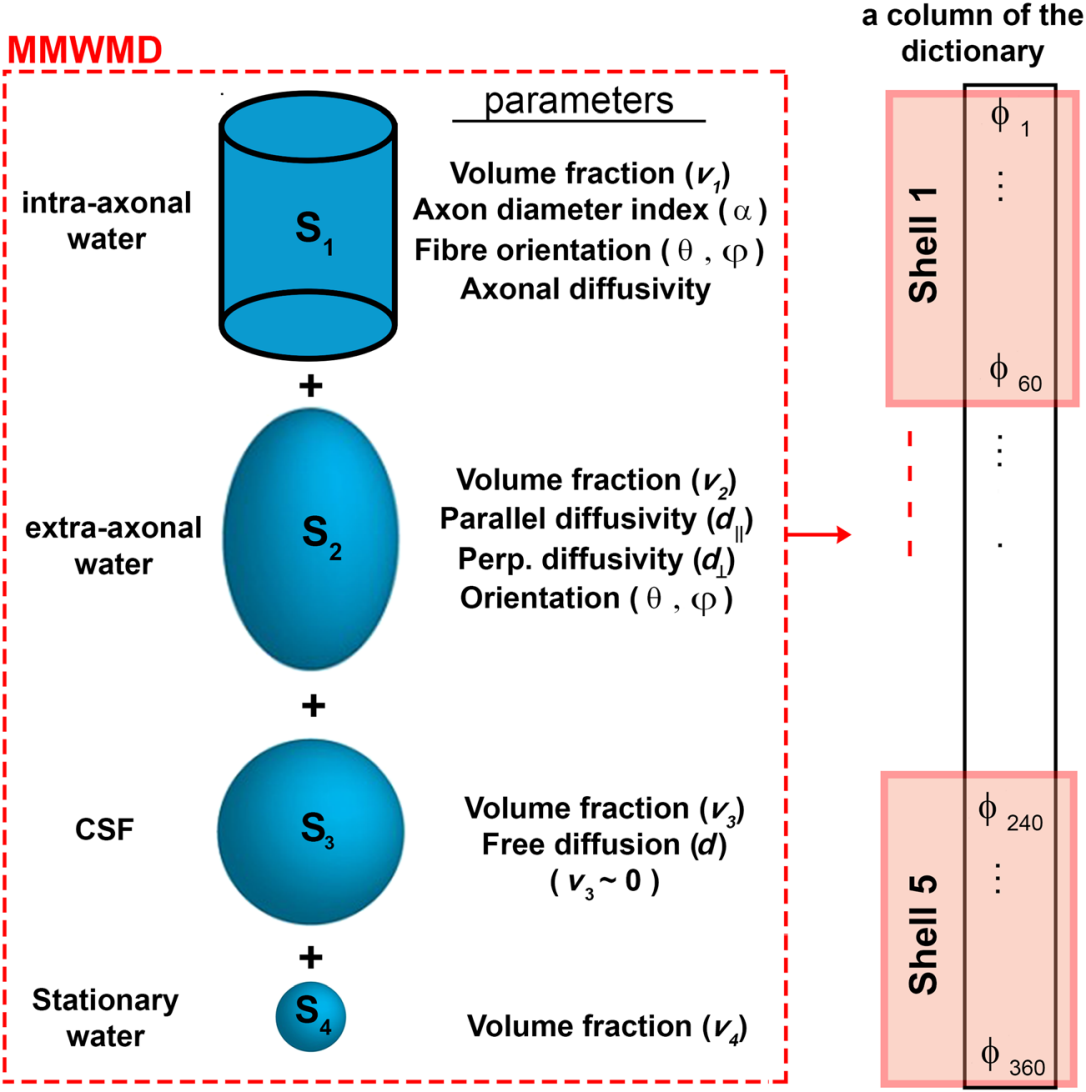
Schematic view of the minimal model of white matter diffusion (MMWMD). Four compartments of the MMWMD model are shown and all parameters of each compartment are presented. An example column of the dictionary in ActiveAx‐D (see ‘ActiveAx‐D’ section) is shown on the right. Each member of the dictionary was generated using the acquisition parameters and a given setting of model parameters. Each column of the dictionary consists of all the diffusion‐weighted measurements in a voxel (360 multi‐shell, higher angular resolution measurements). CSF, cerebrospinal fluid.

To obtain axon diameter indices from diffusion‐weighted data, the MMWMD model 3 was fitted to voxels in brain regions in which the fibre orientation was expected to be homogeneous 3, e.g. the corpus callosum. The three‐stage fitting routine, as described previously 3, 16, was used to obtain the index of the axon diameter for each voxel using the Camino software package 35. MMWMD, the tissue model of ActiveAx, is shown schematically in Fig. 1. All the model parameters are also listed with each component of the model. For each voxel, there are 360 diffusion‐weighted measurements, containing three to five *b* values with different gradient direction encodings (Table 1). A Rician Markov chain Monte Carlo (MCMC) approach was used for model fitting 3. A grid search was first used to identify the starting points, followed by a gradient descent with the same objective function as the grid search, to refine the maximum likelihood parameter estimates. These steps provide a starting point for MCMC, which fixes all the parameters, except the axon diameter index and intra‐ and extra‐axonal volume fractions. Final parameter estimates were calculated from the mean over 2000 iterations. Maps of the index of axon diameter were then drawn for the three‐shell, four‐shell and five‐shell datasets.

### ActiveAx‐D

The index of the axon diameter was obtained for each voxel of the corpus callosum using a dictionary‐based approach, here denoted as ‘ActiveAx‐D’. First, a dictionary was created with the acquisition protocol and the white matter model as input parameters. Then, parameters of interest were extracted from the dictionary. The ActiveAx‐D approach uses the same routine as AMICO, described in detail in ref. 15. In brief, AMICO expresses the ActiveAx model as a linear system that can be formulated as an optimisation problem as follows:

(4) $$\underset{x>0}{\arg\min}\frac{1}{2}{\left\|\Phi x‐y\right\|}_{2}^{2}+\lambda \frac{1}{2}\left|X\left\|{}_{2}^{2}\right.\right.$$

where Φ is a dictionary $\left(\left\{\phi_{i,j}\right\},{\epsilon \mathbb{R}}^{N_{c}\times N_{d}}\right)$, containing *N*_c_ simulated diffusion‐weighted signals obtained from different combinations of MMWMD model parameters and *N*_d_ normalised *q*‐space measurements. $\;x\;{\epsilon \mathbb{R}\;}^{N_{c}}$ are the model parameters to be estimated. $y\;{\epsilon \mathbb{R}\;}^{N_{d}}$ are the *N*_d_ diffusion‐weighted signals in a given voxel of the diffusion‐weighted MRI experiment. ‖⋅‖_2_is the standard *ℓ*_2_ − norm in ${\mathbb{R}}^{n}$. The second term of the optimisation problem, ${\left\|x\right\|}_{2}^{2}$, is included to improve the stability of the estimation 14, and *λ* is the parameter controlling the trade‐off between the data and the Tikhonov regularisation term. Prior to the creation of the dictionary, AMICO fits the DTI model to the data and then creates the dictionary using the estimated fibre orientation.

In AMICO, the dictionary is partitioned into three submetrics, Φ = [Φ^r^|Φ^h^|Φ^i^], where Φ^r^, Φ^h^ and Φ^i^ model the intra‐axonal, extra‐axonal and isotropic water diffusion, respectively. A ‘cylinder‐zeppelin‐ball’ tissue model 14 was adopted to create the dictionary. Each member of the dictionary ({*φ*_*i* , *j*_}), for the *i*th combination of tissue model parameters and the *j*th *q*‐space measurement, can be defined as:

(5) $$\phi_{i,j}=\sum_{k=1}^{4}v_{k}S_{k}\left(t_{i},q_{j}\right)$$

where v_*k*_ is the proportion of water molecules in the *k*th compartment and *S* is the normalised diffusion‐weighted signal, generated from the *i*th combination of model parameters using a tissue compartment model (**t**), and the *j*th measurement using the acquisition pulse sequence (here denoted as **q**).

The differences in formulation between AMICO and ActiveAx‐D include the use of a four‐compartment tissue model, similar to MMWMD, in the latter. Here, the dictionary was defined as:

(6) $$\Phi =\left[\Phi^{r}\left|\Phi^{h}\left|\Phi^{i}\right.\left|\Phi^{s}\right.\right.\right]$$

where Φ^s^ models the stationary water trapped inside small structures. The inclusion of the compartment for stationary water has been suggested previously for *ex. vivo* experiments 3.

A Rician noise model was used when formulating the optimisation problem as the Rice distribution is a more realistic model of noise than the Gaussian distribution in MRI 36, 37. For each shell of the *q*‐space measurement, the SNR was first calculated from the acquired data and Rician noise was added when creating the dictionary. The dictionary can be defined as follows:

(7) $$\begin{array}{l} \Phi =\left[\Phi_{1}\left|\Phi_{2}\left|\ldots \right.\left|\Phi_{q}\right.\right.\right]\\ \Phi_{q}=\left\{\phi_{i.j_{q}}\right\}\in {\text{□}}^{N_{c}\times N_{q}}\\ N_{d}=\sum_{i=1}^{\max\left(q\right)}\mathrm{Nq} \end{array}$$

where *q* is the shell number (e.g. *q*= [1,2,3] for the three‐shell data). Each member of {*ϕ*_*i* , *jq*_} is defined as follows:

(8) $$\phi_{i,j_{q}}=\sum_{k=1}^{4}v_{k}S_{k}\left(t_{i},q_{\mathrm{jq}}\right)+\eta_{q}$$

where *η*_*q*_ is the offset accounting for Rician noise bias in the *q*th shell. Finally, the optimisation problem can be formulated as follows:

(9) $$\underset{x\geq 0}{\mathrm{argmin}}{\left\|\Phi x‐y\right\|}_{2}^{2}$$

### Axon diameter index estimation with ActiveAx‐D

To estimate the axon diameter indices using ActiveAx‐D, a dictionary (Φ) was generated employing the MMWMD model, as explained above. The following inputs were used (a given column of the dictionary is shown in Fig. 1).

1. |*G*|, Δ, δ, TE and gradient directions from the ActiveAx protocol.
2. SNRs of the diffusion‐weighted signals were calculated for each shell of the data. SNR was calculated by measuring *μ*_*q*_/*σ*_*q*_, where *μ*_*q*_ and *σ*_*q*_ are the mean and standard deviation of the diffusion‐weighted signals, respectively, of shell *q* in a given voxel.
3. The fibre orientation and volume fraction of stationary and isotropic water were obtained by fitting the MMWMD tissue model to each voxel from ActiveAx fitting.
4. Axon diameters of 0.1–6 μm in 0.1‐μm steps.
5. Intra‐axonal volume fraction of 0.3–0.9 in 0.05 steps.
6. Parallel diffusivity (*d*_‖_) of 0.6 μm^2^/ms, tortuosity model similar to ref. 3 (tortuosity =(*d*_‖_*v*_2_)/(*v*_1_ + *v*_2_) and zero exchange rate.

The axon diameter index of a given voxel was then extracted by solving Equation (9). The results of ActiveAx‐D were compared with those of conventional ActiveAx and EM across the corpus callosum. Statistical comparisons between techniques (i.e. ActiveAx *versus* ActiveAx‐D) were performed using the paired *t‐*test.

### Region of interest selection

To segment the corpus callosum of the mouse brain, we manually registered the light microscopy image to the *T*_2_‐weighted map, which was obtained from averaging unweighted diffusion images (*B*_0_ images). The corpus callosum and its subregions were manually segmented on the light microscopy image. Then, the mask image obtained was down‐sampled to the dimensions of the diffusion‐weighted images. We only selected the internal voxels in order to exclude non‐callosal voxels.

### Investigation of overestimation

The effect of time‐dependent extra‐axonal diffusion, the sensitivity limitations of the PGSE acquisition protocols and the presence of axonal dispersion were tested as possible explanations for the overestimation of the axon diameter index 3.

#### Time‐dependent diffusion in extra‐axonal water

To ameliorate the effect of non‐Gaussian diffusion in extra‐axonal water on the axon diameter indices, measurements with low *b* values were discarded. Then, the axon diameter indices obtained were compared with estimates from the full dataset. The five‐shell dataset was used for this experiment because the higher number of *b* values allowed data subdivision. We generated a sub‐dataset by discarding the two low *b* value shells (2974 and 3597 s/mm^2^; see Table 1), and compared its derived axon diameter indices with those from the full five‐shell data. A dataset containing only two low *b* value shells was also generated and included in the comparison. MMWMD was then fitted to these datasets using the ActiveAx routine, as explained above in the ‘Axon diameter index estimation with ActiveAx’ section.

#### PGSE pulse sequence limitation

We simulated the signal change for axon diameter indices in the range 0.1–5 μm. All of the simulation parameters were carefully defined to be the same as in our diffusion MRI experiments. The diffusion‐weighted signal was simulated for the gradient direction perpendicular to the axons. The axonal compartment of MMWMD was used to model intra‐axonal diffusion perpendicular to the axon axis, with two different water diffusivities, 0.6 and 1.6 μm^2^/ms, for the simulation.

#### Presence of axonal dispersion

The existence of axonal dispersion was assessed through different investigations. The fractional anisotropy and orientation dispersion index were obtained from diffusion‐weighted MRI. We employed DTI and NODDI to obtain the fractional anisotropy and orientation dispersion index. The shell with the lowest *b* value was used for DTI fitting and the four‐shell dataset was used for NODDI fitting. The Pearson correlation coefficient was also used to measure the linear correlation between fractional anisotropy or neurite orientation dispersion index and estimates of the index of axon diameter in voxels in the mouse corpus callosum.

## Results

### Axon diameter estimates from EM

**Figure 2:**
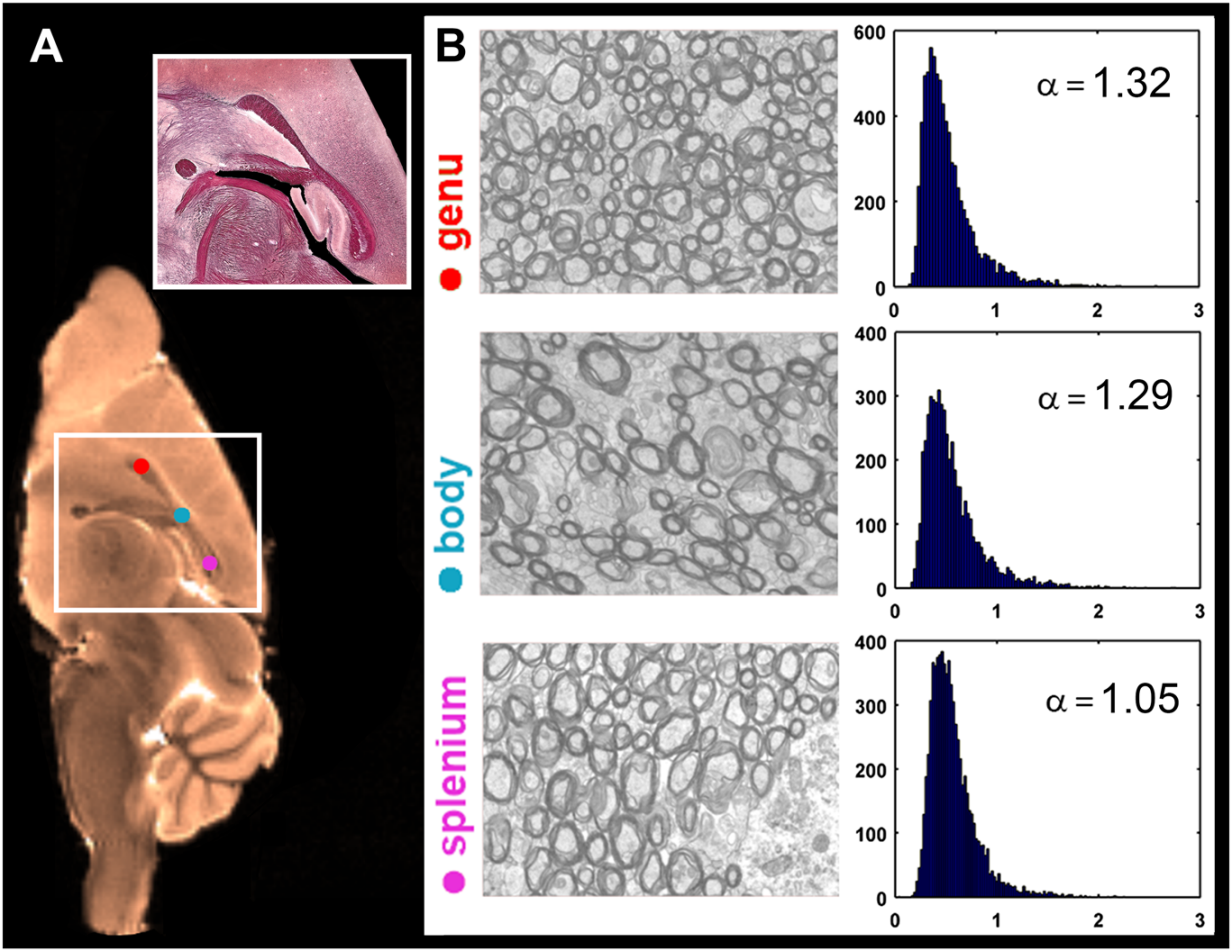
Axon diameter estimates from electron microscopy (EM). (A) *T*_2_‐weighted mid‐sagittal image of the mouse brain (colour‐coded in bronze), together with a light microscopy image of the corpus callosum area (white box). (B) Representative EM images of the corpus callosum subregions and the axon diameter distribution and axon diameter index (α) of each region. The axon diameter indices were obtained from Equation [2]. The diameters of 20 128 axons were measured across the entire corpus callosum: 7680, 5260 and 7188 axons in the genu, body and splenium, respectively.

**Figure 3:**
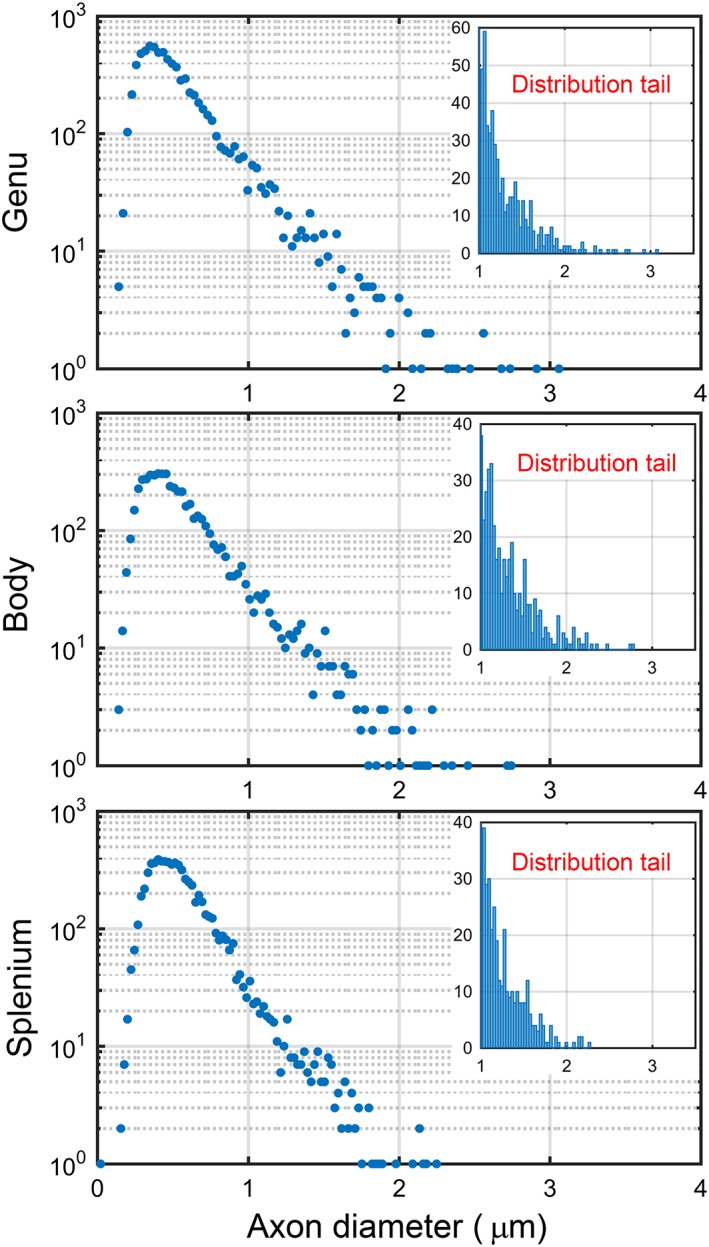
Semi‐logarithmic graphs and tails of axon diameter distributions. For each region of the corpus callosum, a semi‐logarithmic graph of the axon diameter distribution from electron microscopy (EM) was plotted. The axon diameter distributions of axons larger than 1 μm were also included, which represent the tail of the distributions. Diameters of 20 128 axons were measured across the entire corpus callosum: 7680, 5260 and 7188 axons in the genu, body and splenium, respectively.

EM‐derived mean axon diameters and axon diameter indices of regions of the corpus callosum are shown in Figs 2, 3 and Table 2. Measures of axon diameter from EM (i.e. entire corpus callosum: median, 0.49 μm; mean, 0.56 μm) were higher than those reported in a previous study (median, 0.25 μm; mean, 0.43 μm) 38, in which shrinkage artefacts caused by tissue preparation affected the results. We observed relatively uniform values in axon diameter indices of the corpus callosum, with a high–high–low trend across the genu–body–splenium (Table 2, last column).

**Table 2:** Basic statistics of axon diameters of the mouse corpus callosum, obtained from electron microscopy

Region	*n*	Mean ± SD (μm)	Min–max (μm)	Median (μm)	*α* ^a^ (μm)
Genu	7680	0.54 ± 0.28	0.14–3.09	0.47	1.32
Body	5260	0.57 ± 0.29	0.16–2.76	0.49	1.29
Splenium	7188	0.57 ± 0.23	0.03–2.26	0.52	1.05
Whole corpus callosum	20128	0.56 ± 0.27	0.03–3.09	0.49	1.24

a *α* is the axon diameter index [Equation [2]].

SD, standard deviation.

The mean axon diameter in the genu (0.54 ± 0.28 μm) was significantly (*P* < 0.01) smaller than that in the body and splenium (0.57 ± 0.29 and 0.57 ± 0.23 μm, respectively). The mean axon diameter in the splenium was similar to that of the body. The splenium had the highest median value and lowest standard deviation in the axon diameter, the latter reflecting the homogeneity of the axons in this region. The distribution of diameters in the splenium had a comparatively small tail towards higher diameters (Fig. 3). This has also been observed in other mammalian species 39.

### Assessment of the effect of gradient strength

**Figure 4:**
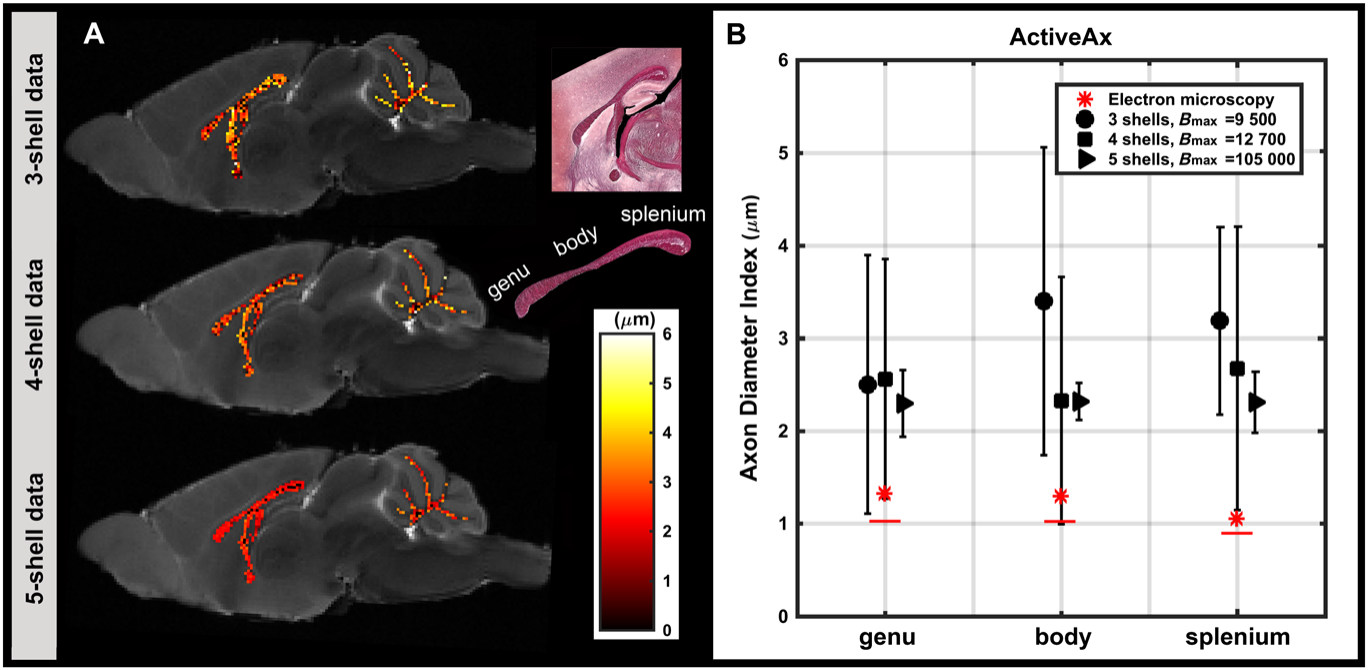
Axon diameter index estimates from ActiveAx. (A) Mid‐sagittal maps of axon diameter index overlaid on the *T*_2_‐weighted image of the mouse corpus callosum, obtained from three‐shell, four‐shell and five‐shell data (see ‘Methods’ section). The mid‐sagittal light microscopy image, covering the corpus callosum area, and an enlarged view of the corpus callosum with its subregions are shown. (B) Mean and standard deviation of axon diameter indices from ActiveAx across subregions of the corpus callosum. Axon diameter indices from electron microscopy (EM) analysis are also included in red. Red stars and red lines are axon diameter indices obtained for wide‐pulse [Equation [2]] and short‐pulse [Equation [3]] limits, respectively.

Figure 4 demonstrates that lower axon diameter indices were obtained with higher *b* values (higher gradient strength), although the axon diameter indices were still overestimated in comparison with EM. In addition, with the highest *b* value (five‐shell data), the estimated values were relatively constant across white matter. The relative homogeneity of the estimated axon diameter index of five‐shell data was in agreement with EM.

For the three‐shell (first row in Fig. 4A) and four‐shell (second row in Fig. 4A) data, considerable variance in the estimated axon diameter indices was observed. However, the variance decreased as we moved to five‐shell data (with higher *b* values). The mean axon diameter index of the body of the corpus callosum was highest when obtained from three‐shell or five‐shell data, but lowest when obtained from four‐shell data.

We observed that the estimated axon diameter indices in the anterior commissure were smallest across the whole sagittal section, where they were all smaller than 1 μm. This may be a result of the high packing density of the aligned fibres in the anterior commissure.

**Figure 5:**
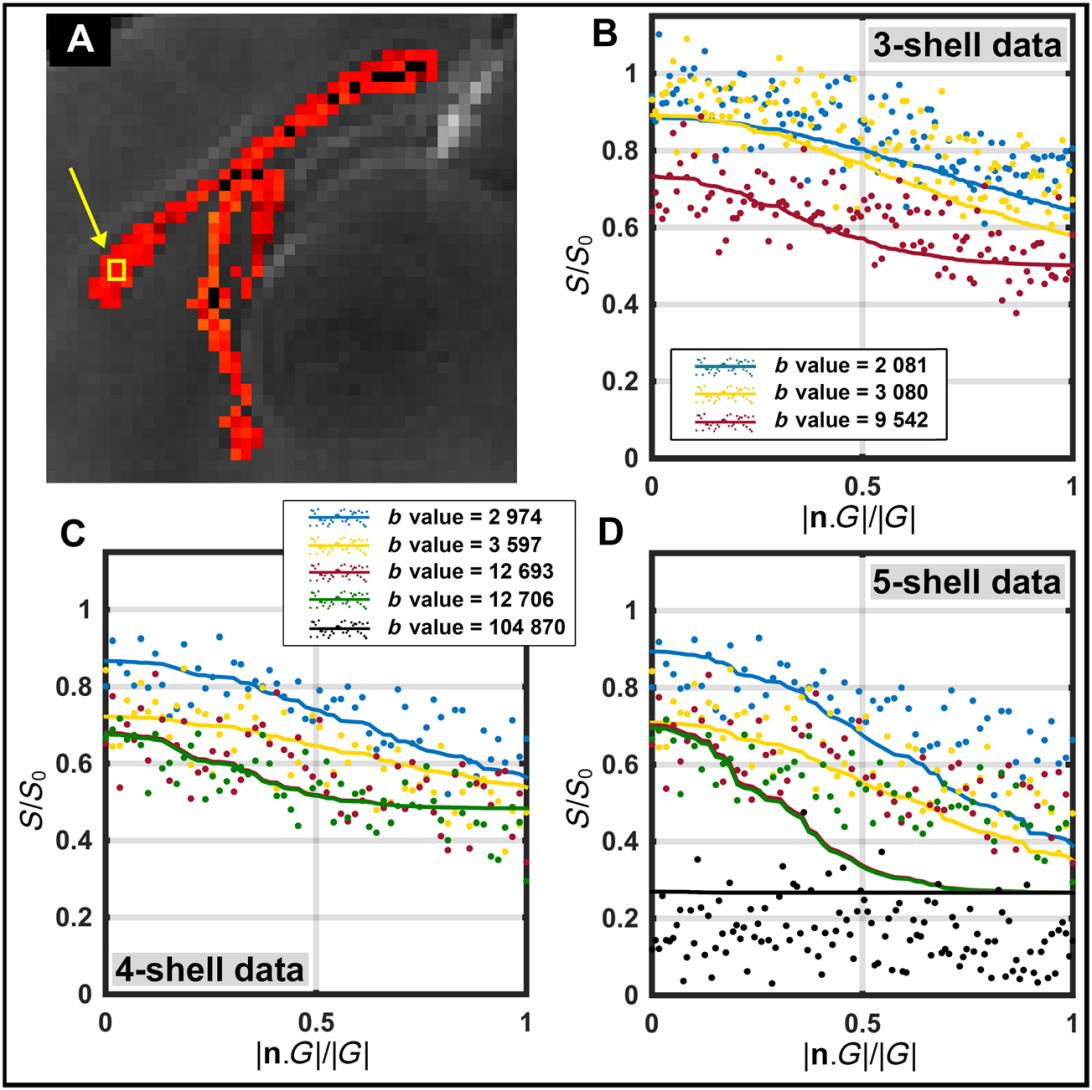
Plots of measurements and predictions from the fitted model in a voxel of the genu. (A) Map of the axon diameter index from five‐shell data (a close up from Fig. A). The example voxel is highlighted with the yellow square/arrow. Measurements and predictions from the fitted model from three‐shell data (B), four‐shell data (C) and five‐shell data (D) are plotted. The full lines show the predicted signals. The gradient directions were aligned with the estimated fibre orientation (*x*‐axis). Measurements were normalised by the unweighted signal (*S*_0_). The *b* values in the legend have the unit of s/mm^2^.

Figure 5 compares the diffusion‐weighted signals with predictions from the fitted model in a voxel of the genu in each of the three‐shell, four‐shell and five‐shell datasets. The presented voxel is typical of callosal voxels in terms of diffusion‐weighted signals and their fits.

The MMWMD model represented the broad trend in the data, especially in the three‐shell and four‐shell datasets. However, in the five‐shell data, it overestimated the measurements of the shell with a *b* value of 105 000 s/mm^2^. In addition, the rate of signal reduction was lower as we moved from a perpendicular to a parallel gradient direction. In the shell with a *b* value of 105 000 s/mm^2^, almost no reduction in signal was observed in the |**n**.*G*|/|*G*| domain (the slope was equal to −0.06 when a line was fitted to high *b* value measurements; for the low *b* value measurements, the slope was −0.23).

### Assessment of the effect of the parameter extraction technique

**Figure 6:**
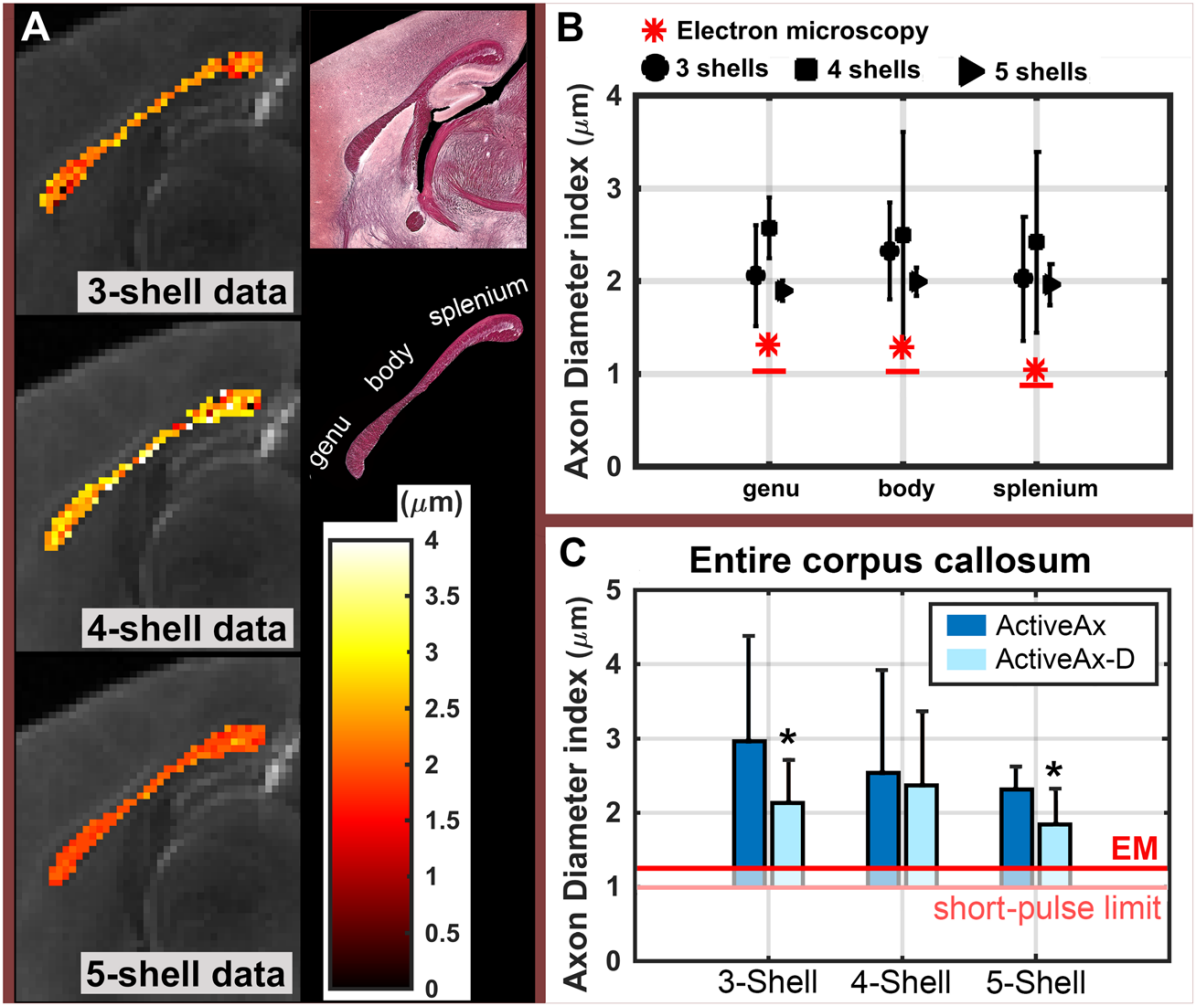
Axon diameter index estimates from ActiveAx‐D. (A) A close‐up of the maps of the axon diameter index overlaid on the *T*_2_‐weighted image for the mouse corpus callosum, obtained from three‐shell, four‐shell and five‐shell data. The mid‐sagittal light microscopy image, covering the corpus callosum area, and an enlarged view of the corpus callosum with its subregions are shown. (B) Mean and standard deviation of the axon diameter indices from ActiveAx‐D across subregions of the corpus callosum. Axon diameter indices from electron microscopy (EM) analysis are shown in red. Red stars and red lines are the axon diameter indices obtained for wide‐pulse [Equation [2]] and short‐pulse [Equation [3]] limits, respectively. (C) Comparison of axon diameter index estimates from ActiveAx and ActiveAx‐D across the voxels of the corpus callosum. Asterisk indicates significant difference using paired *t*‐test (three‐shell, *P* < 0.00001; five‐shell: *P* < 1e‐22). The red line indicates the axon diameter index of the corpus callosum, measured from EM images.

Estimated axon diameter indices for both conventional ActiveAx and ActiveAx‐D were compared with EM values across the regions of the corpus callosum (Fig. 6). Using ActiveAx‐D with three‐shell and five‐shell data, axon diameter indices were more sensitive, i.e. significantly closer to EM estimates, than those obtained from conventional ActiveAx (three‐shell, *P* < 0.00001; five‐shell, *P* < 1e‐22). In addition, a higher stability (lower standard deviation across voxels) was observed in the estimated axon diameter indices. For four‐shell data, the sensitivity and stability were also higher with ActiveAx‐D compared with conventional ActiveAx, but the difference was not significant.

Similar to conventional ActiveAx, the mean axon diameter index of the body of the corpus callosum was highest, only when obtained from three‐shell or five‐shell data. For the five‐shell ActiveAx‐D, the mean values across the three regions of the corpus callosum were the closest estimates to histology compared with the estimates from all other tested datasets and fitting routines.

In general, ActiveAx and ActiveAx‐D overestimated the axon diameter indices by at least 0.6 μm. In our study, the estimated axon diameter indices, obtained from ActiveAx, had a lower bound of ~1.8 μm, regardless of the acquisition protocol and parameter extraction technique.

### Intra‐axonal water fraction

The estimated intra‐axonal water fractions were higher when datasets with higher *b* values were used (Table 3). Intra‐axonal water fractions were around 0.6–0.7 when five‐shell datasets were used. For three‐shell datasets, these values were ~0.45, lower than the expected intra‐axonal water fraction 4, 18, 40. This finding suggests a relationship between the overestimation of axon diameter indices and the underestimation of the intra‐axonal water fraction. As with axon diameter indices, the variation in the estimates of the intra‐axonal water fraction was lower with ActiveAx‐D than with conventional ActiveAx.

**Table 3:** Intra‐ and extra‐axonal volume fraction estimates using different sequences and fitting routines

Dataset	Fitting routine	Volume fraction	
		Intra‐axon	Extra‐axon
Three‐shell	ActiveAx	0.45 ± 0.15	0.24 ± 0.12
	ActiveAx‐D	0.46 ± 0.09	0.10 ± 0.04
Four‐shell	ActiveAx	0.57 ± 0.26	0.11 ± 0.08
	ActiveAx‐D	0.43 ± 0.08	0.12 ± 0.09
Five‐shell	ActiveAx	0.61 ± 0.09	0.13 ± 0.09
	ActiveAx‐D	0.69 ± 0.05	0.11 ± 0.06

### Assessment of possible causes of overestimation

#### Time‐dependent diffusion in extra‐axonal water

**Figure 7:**
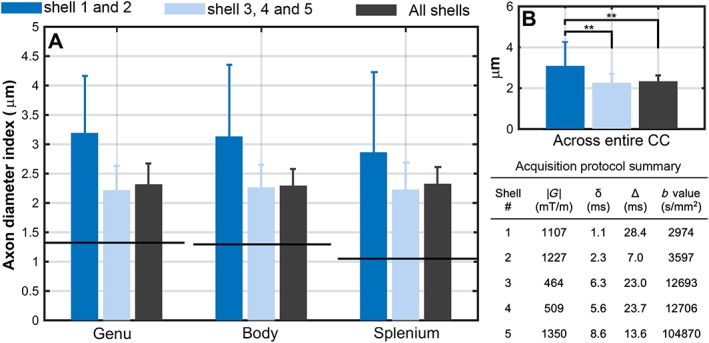
Investigation of overestimation: time‐dependent diffusion in extra‐axonal water. (A) Mean and standard deviation of axon diameter indices from the five‐shell dataset and its subsets. Axon diameter indices from electron microscopy (EM) analysis are shown with a black line [from Equation [2]]. (B) Mean and standard deviation of axon diameter indices across the corpus callosum (CC). A summary of the acquisition protocols of all shells is presented.

No significant difference in axon diameter index estimates was observed when shells with low *b* values were discarded compared with the estimates from the full dataset (Fig. 7). A trend towards possible improvement by discarding low *b* value shells was observed, but could not be confirmed. Both the full dataset (Fig. 7B; black bar) and the dataset with only high *b* value shells (Fig. 7B; light blue bar) yielded significantly lower mean values of the axon diameter indices across the corpus callosum (*P* < 0.01; using paired *t*‐test), when compared with the estimates from the dataset with only low *b* value shells (Fig. 7B; dark blue bar). It should be noted that values from only low *b* value shells were reasonably close to EM values. This may be because of the high gradient strengths employed (>1000 mT/m).

#### PGSE simulation

**Figure 8:**
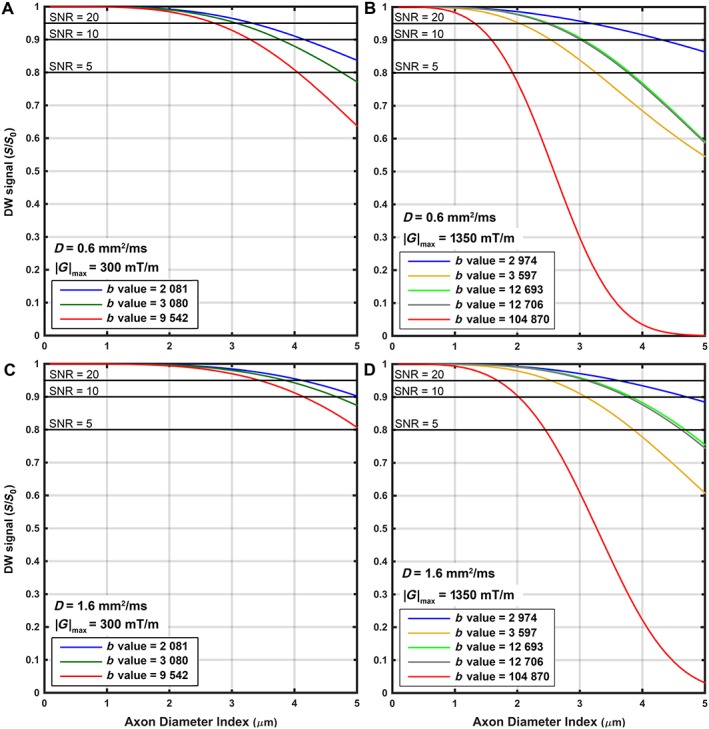
Simulated diffusion‐weighted signal based on the axon diameter index. Simulation was performed based on the pulsed gradient spin echo (PGSE) protocols used in this study. Each line represents a diffusion‐weighted signal change based on the axon diameter index for a given acquisition protocol in Table 1. (A, B) Three‐shell and five‐shell data, respectively at an axonal diffusivity of 0.6 μm^2^/ms. (C, D) Three‐shell and five‐shell data, respectively, at an axonal diffusivity of 1.6 μm^2^/ms. Signal‐to‐noise ratio (SNR) lines were drawn to show the required signal change (attenuation) in order to gain sensitivity to a given axon diameter index. The *b* values in the legend have the unit of s/mm^2^.

Simulations confirm that a greater sensitivity to small axons can be obtained with higher *b* values (Fig. 8), keeping in mind the prominent contribution of the SNR. With *G*_max_ = 300 mT/m and SNR = 20, the diffusion‐weighted signal attenuation is detectable when the axon diameter index is around 2.5 μm or higher (red line in Fig. 8A). When SNR deteriorates to 5, the sensitivity to the small axon diameter decreases (the signal attenuation is detectable when the axon diameter index is at least 4 μm). For *G*_max_ = 1350 mT/m, greater sensitivity can be achieved. Even with SNR of 5, the protocol with a *b* value of 105 000 s/mm^2^ is sensitive to signal changes when the axon diameter index is ~2 μm or higher. With an intermediate *b* value of 3597 s/mm^2^, but using a high gradient strength of 1200 mT/m (orange line in Fig. 8B), the detectable signal change reaches as low as 2 μm when SNR is 20. Figure 8C, D demonstrates that the sensitivity to small axons is lower when an axonal diffusion of 1.6 μm^2^/ms is considered.

In general, the attenuation of the diffusion‐weighted signal was only greater than noise (i.e. detectable) when the axon diameter index was at least 2 μm, given a PGSE pulse with the same imaging protocols as used in our *ex. vivo* imaging experiments.

#### Axonal dispersion in the corpus callosum

**Figure 9:**
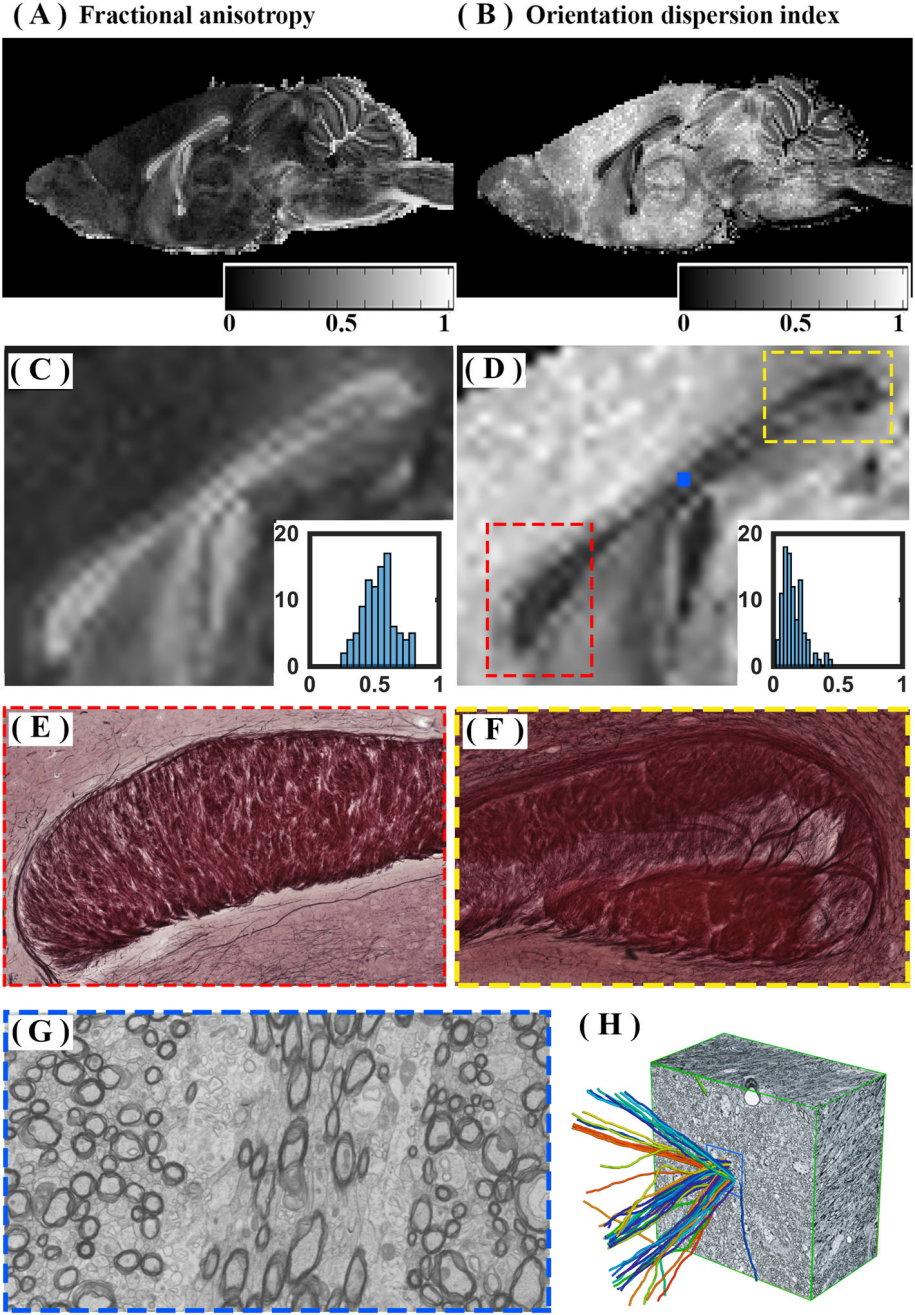
Axonal dispersion in the mouse brain corpus callosum. (A) Mid‐sagittal map of the fractional anisotropy from diffusion tensor imaging (DTI). (B) Mid‐sagittal map of the orientation dispersion index from neurite orientation distribution and density imaging (NODDI). (C, D) Enlarged images of the corpus callosum area from (A) and (B), respectively. Histograms are the fractional anisotropy and orientation dispersion indices across the mouse brain corpus callosum in the mid‐sagittal plane. It should be noted that fractional anisotropy values and orientation dispersion indices were significantly negatively correlated (*r* = −0.7; *P* < 0.01). (E, F) Light microscopy images of the genu and splenium of the corpus callosum. High axonal packing in the genu and relative dispersion in the splenium are observed. (G) Presence of axonal dispersion in the body of the corpus callosum, obtained from electron microscopy (EM). (H) Axonal dispersion in the corpus callosum of a mouse brain [this is fig. 2. c of ref. 41 and is reproduced with kind permission of the journal].

Figure 9 suggests that a large axonal dispersion exists, even in the mid‐sagittal corpus callosum region, which is known to be a bundle of highly packed and aligned fibres. Fractional anisotropy values and orientation dispersion indices were in the ranges of 0.3–0.8 and 0.1–0.5, respectively. A significant negative correlation was observed between the fractional anisotropy and the axon diameter index maps in the corpus callosum (*r* = −0.7, *P* < 0.0001; the higher the fractional anisotropy, the lower the axon diameter index). A significant positive correlation was observed between the orientation dispersion and axon diameter index maps (*r* = 0.34, *P* < 0.001; the higher the neurite dispersion index, the lower the axon diameter index).

Light microscopic images of the genu and splenium, together with a representative EM image from the body of the corpus callosum, are presented for the qualitative assessment of axonal dispersion in Fig. 9E – H. Axonal dispersion is observed in both light microscopic and EM images. In Fig. 9E, F, axonal packing is shown by light microscopy of the genu and splenium, respectively. Although axons in the genu appeared to be packed relatively tightly, axonal dispersion was observed in the splenium. We also observed axonal dispersion in a large number of the EM images. An example is shown in Fig. 9G. Axonal dispersion in the corpus callosum has been demonstrated previously 41. Axonal dispersion can be better appreciated if one employs three‐dimensional histological imaging techniques (Fig. 9H), as in previous block face imaging studies 41.

## Discussion

In this work, we report the non‐invasive measurement of the axon diameter index in the mouse corpus callosum from a diffusion‐weighted MRI acquisition with ultra‐high gradient strength, and validate the measurement against EM data. An *ex. vivo* mouse brain was scanned with a 16.4‐T scanner, allowing imaging with ultra‐high gradient strength and histological validation. The ActiveAx framework with an optimised PGSE pulse sequence at a maximum gradient of 1350 mT/m (maximum *b* value of 105 000 s/mm^2^) was employed to obtain axon diameter indices. Measurements were validated with axon diameter indices obtained from EM in different regions of the mouse corpus callosum. We also compared axon diameter index estimates from ultra‐high gradient PGSE acquisitions with those from a previously tested PGSE pulse sequence using lower gradient strengths 7, with a maximum gradient strength of 300 mT/m.

In brief, we observed that axon diameter indices from sequences with the highest gradient strength were closest to the histology‐derived measures, showing higher sensitivity to small diameter axons compared with a previously tested sequence 7. Regardless of the improved sensitivity, we observed overestimation in the obtained axon diameter indices of the mouse corpus callosum compared with EM values (at least 0.6 μm; Figs 4, 6). Further analysis of the pulse sequence and the tissue compartment models suggests that the limitations of the PGSE pulse sequence and the complexity of brain tissue microstructure, in particular axonal dispersion, are probable contributors to the overestimation. In addition, we adapted the axon diameter index formulation as defined in ref. 18 to obtain axon diameter indices from EM images, resulting in less overestimation than in previous work 3.

### Gradient strength

Using the ultra‐high gradient strength pulse sequence (*G*_max_ = 1350 mT/m), significantly higher sensitivity to axon diameter indices (~2 μm lower bound; Fig. 4) was found compared with the data obtained with *G*_max_ = 300 mT/m (~3 μm lower bound; Fig. 4). A homogeneous axon diameter index map was obtained across the corpus callosum (Fig. 4), which followed a similar trend to the values from EM (Figs 2, 3). From EM images, characteristics of the axon diameter distribution in different subregions of the mouse corpus callosum were replicated, with a shorter tail of the distribution in the splenium, and a longer tail of the distribution in the body. A similar pattern was observed in the corpus callosum of the rat in the study by Barazany *et al*. 4. However, unlike the present study, they also observed a difference between the axon diameter distribution of the body of the corpus callosum compared with the splenium and the genu.

The quality of fit in the five‐shell data (*G*_max_ = 1350 mT/m) was poorer than for three‐shell and four‐shell datasets (Fig. 5). The effect of noise and the simplicity of the model are likely to be the reasons for this. High *b* value measurements in the five‐shell data may hit the noise floor. In addition, ActiveAx does not model permeability and uses a simple tortuosity model for extra‐axonal water displacement, which may not hold for such high *b* value acquisitions.

### Parameter extraction routine

The sensitivity to axon diameter indices was higher for the dictionary‐based approach (ActiveAx‐D), for datasets with G_max_ = 300 mT/m and *G*_max_ = 1350 mT/m, compared with conventional ActiveAx (Fig. 6C). The dictionary‐based approach used here is similar to the recently proposed AMICO technique 15, which appeared during the preparation of the manuscript. The key differences between ActiveAx‐D and AMICO are as follows.

1. Unlike AMICO, which assumes a Gaussian noise distribution, ActiveAx‐D uses a Rician noise model, based on SNR values measured from the data and incorporated in the creation of the dictionary. Our SNRs were calculated as the mean of the diffusion‐weighted signal divided by the standard deviation, and may overestimate the SNR. The fitting of a comprehensive noise model may provide a more accurate estimate of SNR than we have been able to achieve in this study.
2. Although AMICO is a framework somewhat independent of the choice of model, the ActiveAx‐D application here uses an additional compartment compared with the AMICO experiments in ref. 15 to account for stationary water, as in ref. 3.
3. AMICO [at least the application in ref. 15] fits the DTI model to the data prior to creating the dictionary, whereas ActiveAx‐D uses a four‐compartment model, similar to NODDI. As a result, AMICO has a shorter computation time than ActiveAx‐D. However, the primary aim of ActiveAx‐D was to improve the sensitivity and stability of the estimation of axon diameter indices.

ActiveAx‐D includes the noise model when creating the dictionary, which relaxes the need to extract model parameters with ridge regression approaches, such as Tikhonov regularisation used in AMICO. Ordinary least‐squares methods are employed to solve the linear regression model in ActiveAx‐D [Equation ((9))]. It is possible that adopting this approach could decrease the computation time of AMICO further, and future work may combine the favourable aspects of both techniques for further improved parameter estimation.

### Intra‐axonal water volume fraction

**Figure 10:**
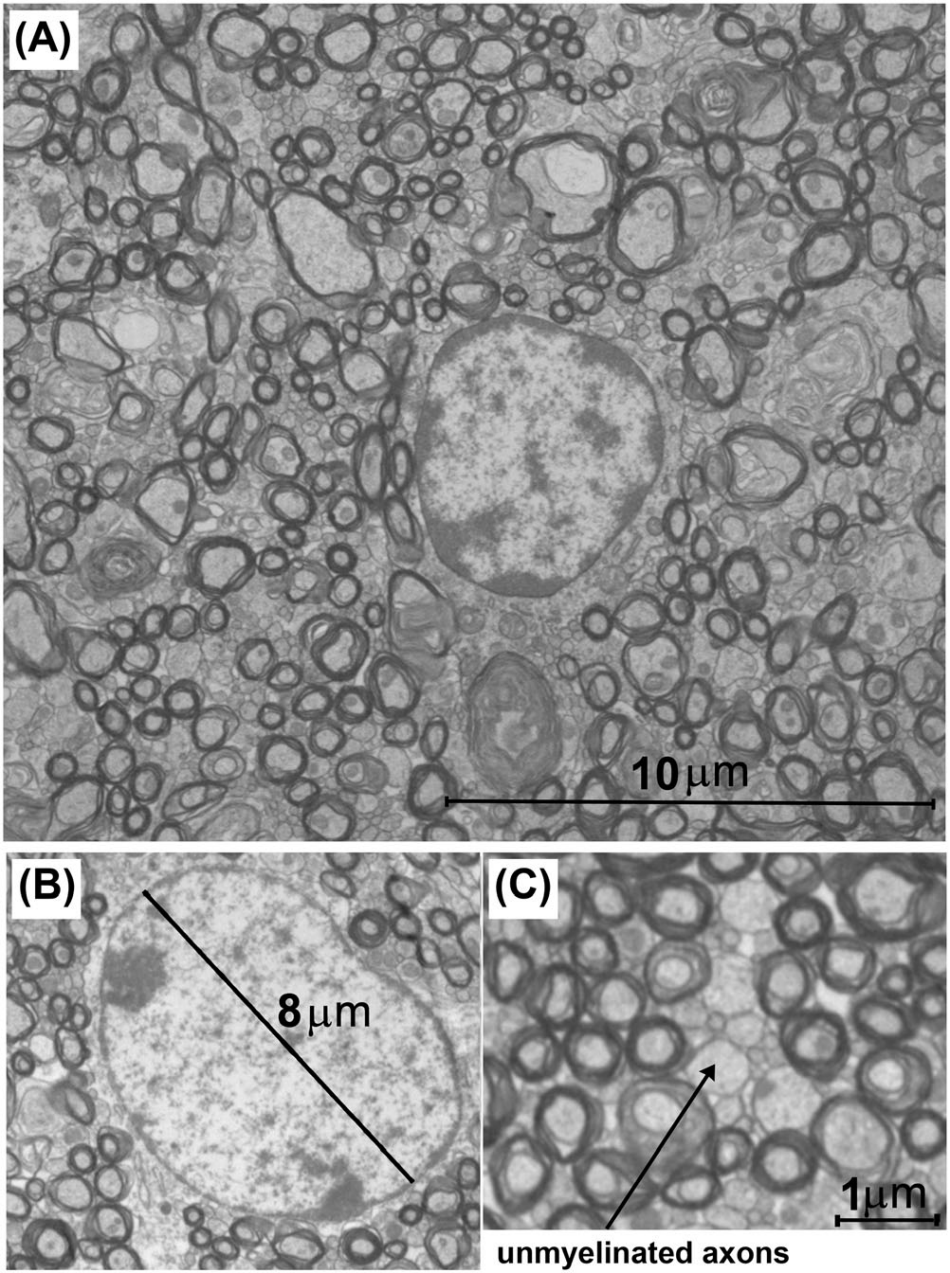
Glia cells and extra‐‘myelinated axonal’ space. (A) A 20 × 20‐μm^2^ electron microscopy (EM) image from the genu of the corpus callosum. Note that the diameter of the oligodendrocyte glial cell in the middle of the image is much larger than the axon diameters. (B) Another example of an oligodendrocyte from the genu of the corpus callosum. (C) A zoomed view of a region of the corpus callosum with a low density of myelinated axons. Note that this region is packed with unmyelinated axons and neurite processes, and that the diffusion of extra‐axonal water is highly restricted in short time frames.

Histological analyses revealed a high packing density of axons in the corpus callosum. Consequently, a high volume fraction of intra‐axonal water (~0.7–0.8) is expected 4, 18, 40. Our intra‐axonal water volume fraction estimates from five‐shell data support this expectation (~0.6–0.7). For the three‐shell dataset, however, the volume fraction was underestimated (~0.45), which may reflect additional sensitivity to intra‐axonal water with high gradient strength, but may also be spurious as the model fits the high *b* value data poorly. An accurate estimation of the intra‐axonal water fraction from histology images requires accurate estimates of the extra‐axonal water fraction. With the current resolution and contrast in our histological images, it is difficult to distinguish extra‐axonal water from unmyelinated axons, limiting the accuracy with which the extra‐axonal water fraction can be estimated from EM (e.g. Fig. 10C).

Our model assumption regarding intra‐axonal water diffusivity (0.6 μm^2^/ms) affects the volume and diameter estimates. With faster water diffusion, the effects of restriction are stronger, and therefore sensitivity to small axons is lower. Our simulations (Fig. 8) showed that, with a higher diffusivity value (1.6 μm^2^/ms), the sensitivity to the axon diameter index was diminished, with the lower bound increasing from ~1.5 μm to ~2 μm for the shell with the highest *b* value at an SNR of 10.

### Overestimation

We studied three probable reasons for the overestimation of the axon diameter index: presence of time‐dependent diffusion in extra‐axonal water; limitations of the PGSE pulse sequence; and axonal dispersion.

#### Time‐dependent diffusion of extra‐axonal water

A decrease in the estimates of the axon diameter index (not statistically significant) was observed when diffusion‐weighted measurements with low *b* values were discarded (shells 1 and 2 of five‐shell data; Table 1). A larger variance was also observed in the axon diameter estimates of the sub‐datasets, which most likely relates to the lower number of *b* values used. Our results suggest a possible improvement by discarding low *b* value shells, but remain insufficient to confirm the effect of the time‐dependent diffusion of extra‐axonal water. Further experiments with dense sampling of the *b*‐value space are required for a more systematic investigation of these initial observations. For example, a dataset similar to a recent study by Ferizi *et al*. 42 could allow such an investigation, given the high sampling rate in *q* space, but this is beyond the focus of this paper.

The potential benefit of discarding low *b* value measurements requires further investigation as the current suggested models do not capture the complexity of white matter microstructure. The time‐dependent diffusion of extra‐axonal water may be applicable to the detection of mesoscopic structure in white matter 17, potentially a beneficial biomarker for pathological changes, such as demyelination. More recently, it has been suggested that the logarithmic singularity arising from extra‐axonal non‐Gaussian diffusion in short time frames (~*t*_0_) biases axon diameter estimates. At long diffusion times (*D*_*∞*_), non‐Gaussian diffusion has a minimal effect on the intra‐axonal signal change detected. A power spectrum analysis of an EM image from the mouse corpus callosum [fig. 3 of ref. 18] supports a tissue structure (e.g. axonal packing) compatible with logarithmic time‐dependent diffusion of extra‐axonal water. The power spectrum was calculated from a binary image, ‘1’ when a pixel was located inside myelinated axons and ‘0’ elsewhere, which is a simplified (if not oversimplified) presentation of white matter tissue. Such a model does not take into account the highly packed unmyelinated axons and cellular processes, segmenting them as extra‐axonal space (‘0’ in the binary image). Figure 10C shows that, in addition to the space occupied by myelinated axons, unmyelinated axons and glial processes also contribute to highly restricted diffusion over a short range.

We observed a large number of glial cells of 5–10 μm in diameter, such as oligodendrocytes, in our EM images (Fig. 10A, B). Water diffusion within these cells is of possible importance, but was not considered in ref. 18. Intracellular diffusion in these cells is not restricted to a short range and could bias the diffusion estimates of extra‐axonal water. The extent of potential bias depends on the relative volume fraction of glial cells compared with the rest of extra‐axonal space. Our EM images indicate that, because of high axonal packing (myelinated and unmyelinated), extra‐axonal density in the corpus callosum is relatively low. Neglecting the water diffusion in glial cells may thus heavily influence the measurements of extra‐axonal short‐range water diffusion and warrants further investigation. It should be noted that MMWMD does not explicitly include this compartment. The stationary water compartment in MMWMD has been suggested to reflect water trapped in glial cells 3, but this does not correspond well with our EM observations. The inclusion of a stationary water compartment in *ex. vivo* ActiveAx was shown to be beneficial 3, but this is likely to be for other reasons, such as compensation for the bias caused by the presence of fixation solution.

#### Limitations of the PGSE pulse sequence

Simulation of the PGSE pulse sequence showed that, for the sequence with *G*_max_ = 300 mT/m, the attenuation of diffusion‐weighted measurements was not detectable (substantially smaller than noise) when an axon diameter index of 1 μm was considered (Fig. 8A). The minimum values of the axon diameter index at which the attenuation of the diffusion‐weighted measurement was larger than noise were around 2.5 μm for a realistic SNR value (SNR of 20). Sensitivity is dramatically higher with *G*_max_ = 1350 mT/m (Fig. 8), even with a low SNR. In general, simulation showed that, with the use of a PGSE pulse, with *G*_max_ as high as 1350 mT/m, the attenuation of diffusion‐weighted measurements was only detectable (larger than noise) for axon diameter indices of ~2 μm or higher for SNR of 20 (~1.5 μm or higher for SNR of 10). The insensitivity of PGSE to small axons could partly explain the observed overestimation of axon diameter indices in our *ex. vivo* experiment.

#### Presence of axonal dispersion

We also observed a relatively large amount of axonal dispersion even in the corpus callosum (Fig. 9). Axonal dispersion affects axon diameter index estimation for the MMWMD model, as water diffusivity in the estimated perpendicular direction is not homogeneous in all axons 43. MMWMD models the axonal compartment as a pack of aligned cylinders, leading to the overestimation of the axon diameter index in the presence of axonal dispersion.

### Applications

The most practical outcome of our study relates to the obtained improvement in axon diameter index estimation with *G*_max_ = 300 mT/m; such gradient strengths are now available because of the extensive efforts of the researchers involved in the human connectome project 6. Gradients of this magnitude yield a sensitivity down to around 2–3 μm, which offers some potential utility for human studies. Lower SNR would be expected for *in vivo* human brain imaging compared with our *ex. vivo* experiment, and would affect the accuracy of the obtained values.

With the high *b* value acquisition protocols used here, axon diameters as small as ~1 μm were obtained in some voxels, e.g. anterior commissure. Such protocols are impractical for *in vivo* human studies, but could be used to study autopsy and biopsy tissues. In addition, the sensitivity to small axons may be lower for *in vivo* applications, because of cardiac pulsatile movements of the microvasculature and ventricular structures of the brain.

### Limitations and considerations

Corroborating previous studies, we observed that the use of a high gradient strength is highly beneficial for microstructural imaging 7, 44. However, the low SNR of sequences with a *b* value of 105 000 s/mm^2^ limits the achievable sensitivity to small axons (Fig. 8). A similar sensitivity was obtained with an optimised protocol with lower *b* values and higher SNR. Therefore, improving the SNR, by modification of the acquisition set‐up (e.g. by acquiring repeated measurements) or by the application of post‐processing de‐noising techniques, such as lop‐diffusion‐weighted imaging 45, is likely to be beneficial.

In addition to gradient strength, other aspects of the pulse sequence, such as the pulse duration and diffusion time (*δ*, *Δ*), are important to increase sensitivity and stability. Therefore, an optimised, model‐based, pulse sequence, such as ActiveAx 3, 16, is desirable to maximise performance. Here, we used the pulse sequence optimisation routine explained in refs. 3, 16.

The performance of the four‐shell dataset was unsound compared with the other datasets. Axon diameter indices, obtained from the four‐shell dataset, had low stability (high standard deviation). When ActiveAx‐D was employed, the axon diameter indices from this dataset were higher than those from the three‐shell dataset, which had lower gradient strength. The four‐shell dataset was generated as a subset of an optimised pulse sequence and it is possible that the subset was not optimal, accounting for the relatively poor performance of the four‐shell data.

### Future work

Future studies focusing on the utilisation of highly sensitive pulse sequences, advanced parameter extraction techniques and a comprehensive tissue model could increase the accuracy of axon diameter mapping. For example, in pulse sequencing, recent studies have suggested that oscillating gradient spin echo (OGSE) may lead to higher sensitivity to small pores compared with PGSE, at least in the presence of orientation uncertainty and dispersion 46, 47, 48, 49, 50. In contrast, a simulation study by Burcaw *et al*. 18 predicted that OGSE may have lower sensitivity to small pores, because of the relatively higher frequency dependence of the extra‐axonal space compared with the intra‐axonal space. These conclusions were chiefly based on simulation studies using different tissue models. Further investigation is required to fully assess the value of OGSE on probing small pores. For parameter extraction, an advanced machine learning approach, such as random forest regression, could be used in the training stage of ActiveAx‐D to enable parameter extraction between the columns of the dictionary [see, for example, ref. 51]. Finally, a higher sensitivity could be obtained by employing comprehensive tissue models, for example based on Monte Carlo simulations 51, and incorporating additional characteristics, such as fibre dispersion and permeability. It should be noted that the latter would be at the expense of an increase in the number of unknown parameters, and therefore run the risk of decreased stability.
